# Clinical diagnosis and treatment of seven patients diagnosed pneumonia caused by *Chlamydia abortus*: a case series report

**DOI:** 10.3389/fmed.2024.1406737

**Published:** 2024-06-11

**Authors:** Ya Wen, Yanjia Du, Xiaoyan Shi, Zixiong Zeng

**Affiliations:** Department of Pulmonary and Critical Care Medicine, Meizhou People's Hospital, Meizhou, Guangdong, China

**Keywords:** *Chlamydia abortu*s, pneumonia, bronchoalveolar lavage, blood, metagenomics next-generation sequencing

## Abstract

**Background:**

*Chlamydia abortus* pneumonia is very rare in normal people. At present, there is a lack of clinical data on the clinical characteristics and diagnosis and treatment experience of patients with this type of infection. Our team had recently treated 7 cases of these patients. This study aims to comprehensively summarize and analyze the clinical characteristics and treatment methods of *Chlamydia abortus* pneumonia, and to provide clinical evidence for the diagnosis and treatment of *Chlamydia abortus* pneumonia.

**Methods:**

Clinical data were retrospectively collected from patients diagnosed with *Chlamydia abortus* pneumonia through metagenomic next-generation sequencing (mNGS) at the Department of Pulmonary and Critical Care Medicine, Meizhou People’s Hospital.

**Results:**

Seven patients with *Chlamydia abortus* pneumonia reported a history of poultry exposure, experiencing fever alongside respiratory or digestive symptoms. Marked elevation of blood inflammation markers, accompanied by hypoproteinemia and liver damage, was observed. Chest CT scans revealed pneumonia and pleural effusion. *Chlamydia abortus* was detected in blood or bronchoalveolar lavage fluid (BALF) through mNGS, often co-occurring with *Chlamydia psittaci* or other bacteria infections. Notably, Doxycycline demonstrated efficacy in treating *Chlamydia abortus.*

**Conclusion:**

*Chlamydia abortus* infection is a zoonotic disease, particularly among individuals with a history of poultry exposure, and mNGS emerges as a reliable diagnostic tool for its detection. *Chlamydia abortus* infection manifests with systemic and lung inflammation, effectively addressed through Doxycycline therapy.

## 1 Introduction

Chlamydia, a Gram-negative bacterium prevalent in animal cells, can be transmitted to humans through contact with infected poultry or mammals, making it a zoonotic microorganism (1). Various Chlamydia species can infect humans, including *Chlamydia pneumoniae*, *Chlamydia psittaci* (2), *Chlamydia trachomatis*, *Chlamydia abortus*, etc. *Chlamydia pneumoniae* is the most common form of chlamydia that infects the human respiratory tract (3). *Chlamydia trachomatis* is one of the most common microorganisms for human venereal infections (4). Recent years have seen a higher prevalence of clinical cases linked to *Chlamydia psittaci*. This could be attributed to both the widespread use of mNGS detection technologies and increased exposure of farm animals to animal reservoirs, due to habitat destruction and thus increased zoonotic risk. Despite deepening research on *Chlamydia psittaci*, systematic epidemiological survey data are still lacking. Human infection with *Chlamydia psittaci* can result in severe lung consolidation, systemic inflammatory reactions, and liver impairment (5, 6).

While *Chlamydia abortus* infection is rare in the general population, it poses a risk to pregnant women (7), potentially leading to miscarriage or severe acute respiratory distress syndrome. However, cases of *Chlamydia abortus* infection are infrequently reported in non-pregnant individuals (8, 9). In 2023, two case reports from China have highlighted clinical characteristics of *Chlamydia abortus* infection (10, 11) indicating an increased incidence of human infection with *Chlamydia abortus* compared to previous observations. In the past 6 months, our hospital has diagnosed seven cases of *Chlamydia abortus* infection. This study aims to retrospectively analyze the clinical characteristics, diagnosis, and treatment of these seven patients, providing valuable clinical evidence for the management of *Chlamydia abortus* infections.

## 2 Methods

### 2.1 Study design and patients

We conducted a retrospective analysis of all patients diagnosed with pneumonia caused by *Chlamydia abortus* through metagenomic next-generation sequencing (mNGS) in the Department of Pulmonary and Critical Care Medicine at Meizhou People’s Hospital from July 2023 to November 2023. The clinical data, test and examination results, and treatment methods were extracted from the Hospital Information System (HIS). The study was approved by the Meizhou People’s Hospital Ethics Committee [2023-C-111].

### 2.2 Clinical data collection

Basic information: gender, age, inpatient department, time of admission, time of discharge, length of hospital stay, underlying diseases, contact history, smoking history, body mass index (BMI), symptoms, peak body temperature.

Examination report: arterial blood gas analysis (PH, PaO_2_, PaCO_2_, oxygenation index), blood routine (WBC, NE%, L%, Hb), plasma D-dimer, blood tests (CRP, IL-6, PCT, albumin, globulin, aspartate aminotransferase, alanine aminotransferase, creatinine, creatine kinase, lactate dehydrogenase, Na, Ka), chest CT scans (before and after treatment), mNGS examination (Guangzhou Huayin Medical Laboratory Center Co., Ltd), tracheoscopy (lavage tracheal segment).

Treatment: anti-infective regimen for patients before and after diagnosis.

### 2.3 Statistical analysis

Statistical analysis was performed using SPSS 23.0 (IBM, Armonk, NY, United States). Continuous variables were presented as mean ± standard deviation (SD). Categorical variables were expressed as frequencies with percentages.

## 3 Results

### 3.1 Patients demographics

Among the seven patients, five were male and two were female, with ages ranging from 54 to 79 years (average age of 67.14 ± 8.17 years). Four male patients had a history of smoking. All patients reported a history of exposure to poultry, encompassing chickens, ducks, and geese. Six patients had a normal BMI, while one was classified as obese. Two patients had hypertension, two had type 2 diabetes, and one patient with obesity developed fatty liver. All seven patients experienced fever symptoms: six patients had cough and expectoration; three patients had dyspnea; one patient had hemoptysis; one patient had bellyache and nausea; and one patient had abdominal distention. The highest recorded peak fever in seven patients was 40.3°C. The mean length of hospital stay was 7.86 ± 4.67 days (Table 1).

**Table 1:** Basic information of the patients.

Case	1	2	3	4	5	6	7
Gender	Male	Male	Male	Male	Female	Male	Female
Age	69	64	62	74	68	54	79
History of smoking	Y	Y	Y	N	N	Y	N
History of exposure	Poultry	Poultry	Poultry	Poultry	Poultry	Poultry	Poultry
BMI	21.0	22.0	21.8	21.8	22.5	33.3	21.5
Underlying disease	Paroxysmal atrial fibrillation	Hypertension;Type2 diabetes	/	Brain atrophy; Cerebral arteriosclersis	Type2 diabetes; Anaemia	Hypertension	Fatty liver; Aortic sclerosis
Symptom	Cough; dyspnea; fever	Cough; expectoration; fever;dyspnea	Cough; expectoration; fever; dyspnea	Fever; bellyache; nausea	Fever; cough	Fever;cough; hemoptysis; abdominal distension	Cough; expectoration; fever
Temperature spikes	40.3	39.0	40.3	39.4	39.2	40.0	40.0
Hospital stay	18	6	7	8	5	7	4

### 3.2 Laboratory findings

Arterial blood gas analysis revealed hyperventilation in six patients, hypoxemia in three patients, and an oxygenation index below 300 in five patients. Blood cell analysis of seven patients showed that five patients had normal leukocytes. All patients had a higher proportion of neutrophils, and a lower proportion of lymphocytes. Six patients had albumin less than 30 g/L. Plasma D-dimer was elevated in five patients. Five patients had abnormal liver function. The blood levels of LDH, CRP, IL-6 and PCT were all elevated in seven patients. Serum potassium was below normal in five patients (Table 2).

**Table 2:** Patient’s laboratory findings.

Case	1	2	3	4	5	6	7
PH (7.35–7.45)	7.470	7.510	7.450	/	7.503	7.504	7.462
PaO_2_ (80-100 mmHg)	93	73	122	/	56.6	60.6	94.8
PaCO_2_ (35-45 mmHg)	27	21	28	/	27.3	25.3	25.9
PaO_2_/FiO_2_	226.3	178.7	583.1	/	195	289	256
WBC (3.5–9.5 × 10^9^/L)	12.11	11.61	9.92	10.3	9.2	6.2	9.5
NE (45–75%)	93.4	95.1	77.4	88.2	87.1	80.7	75.1
LYMPH (20–50%)	4.5	2.4	13	4	9.9	10.5	16
ALT (7-40 U/L)	36	71.1	52	49	29	48	13
AST (13-35 U/L)	41.9	113.2	51	61	29	43	19
CRE (35-80umol/L)	92	76.8	117.4	82	62	85	78
CK (24-170 U/L)	208.1	1188.1	153	204	28	462	48
CK-MB (0-25 U/L)	10.1	46.2	12.3	14	2.4	26	12.1
LDH (109-245 U/L)	253.6	420.7	210.8	375	202	260	204
ALB (40-55 g/L)	32.8	27.4	33.4	29.3	24.1	38	32.6
GLB (20-40 g/L)	27	25.2	28.1	25.8	29.5	30.2	27.2
CRP (mg/L)0–6	357.75	97.8	63.34	182.5	216.3	203.1	181.5
IL-6 (<7 pg/mL)	277.76	78.46	192.01	/	91.31	84.53	/
PCT (0–0.10 ng/mL)	8.23	2.18	0.07	0.92	0.33	0.26	0.14
Na (137-147 mmol/L)	139.4	134	134.6	133	134	133	134
K (3.5–5.3 mmol/L)	2.97	3.66	3.34	3.89	2.97	2.99	3.39
D-Dime (0–0.5 mg/L)	3.73	3.96	/	/	2.84	2.58	1.35

### 3.3 The patient’s mNGS findings

Among the seven patients, six underwent mNGS analysis with BALF obtained through the bronchial segment of the subtracheal bronchoalveolar lavage lesion, while one patient had mNGS testing conducted on blood samples. The mNGS results revealed *Chlamydia abortus* infection in all seven patients. Among them, four patients had a single infection, two patients were co-infected with *Chlamydia psittaci*, and one patient was co-infected with *Chlamydia psittaci* and *Pseudomonas aeruginosa* (Table 3).

**Table 3:** The patient’s mNGS test results.

Case	Sample	The site of alveolar lavage	Genus			Species	
			Name	Sequence number	Relative abundance%	Name	Sequence number
1	BALF	/	*Pseudomonas*	1,501	0.41	*Pseudomonas aeruginosa*	1,101
			*Chlamydia*	99,851	27.52	*Chlamydia abortus*	41,388
			*Chlamydia*	99,851	27.52	*Chlamydia psittaci*	30,005
2	BALF	Basal segment of the right lower lobe	*Chlamydia*	2,180	0.43	*Chlamydia abortus*	1,474
3	BALF	Lateral basal segment of the right lower lobe	*Chlamydia*	244	18.76	*Chlamydia abortus*	110
			*Chlamydia*	244	18.76	*Chlamydia psittaci*	72
4	Blood	/	*Chlamydia*	18	4.12	*Chlamydia abortus*	7
5	BALF	Left dorsal lower lobe	*Chlamydia*	2,834	74.05	*Chlamydia abortus*	1,172
			*Chlamydia*	2,834	74.05	*Chlamydia psittaci*	856
6	BALF	Left upper lobe apical segment	*Chlamydia*	28	18.54	*Chlamydia abortus*	17
7	BALF	Medial anterior basal segment of the right lower lobe	*Chlamydia*	959	3.90	*Chlamydia abortus*	667

### 3.4 Anti-infective regimen and treatment details

All patients received empiric anti-infective therapy, including β-lactam or β-lactam/lactamase inhibitors and Quinolones. And case one was treated with Meropenem for severe infection. Following diagnosis, all patients were administered Doxycycline, among which case one received Omadacycline due to poor treatment effect (Table 4). Subsequent to Doxycycline treatment, significant improvement was observed in the remaining patients. Additionally, three patients received hepatoprotective therapy, and one patient underwent hemostasis.

**Table 4:** Anti-infective regimen before and after diagnosis of the patients.

Case	Antibiotic regimen	
	Before diagnosis	After diagnosis
1	Moxifloxacin, Piperacillin tazobactam, Meropenem	Doxycycline, Omadacycline
2	Piperacillin tazobactam	Doxycycline
3	Moxifloxacin, Piperacillin tazobactam	Doxycycline
4	Cefmetazole, Piperacillin tazobactam, Metronidazole, Levofloxacin	Doxycycline
5	Piperacillin tazobactam	Doxycycline
6	Moxifloxacin	Doxycycline
7	Piperacillin tazobactam	Doxycycline

### 3.5 Chest CT scans before and after treatment

**Figure 1:**
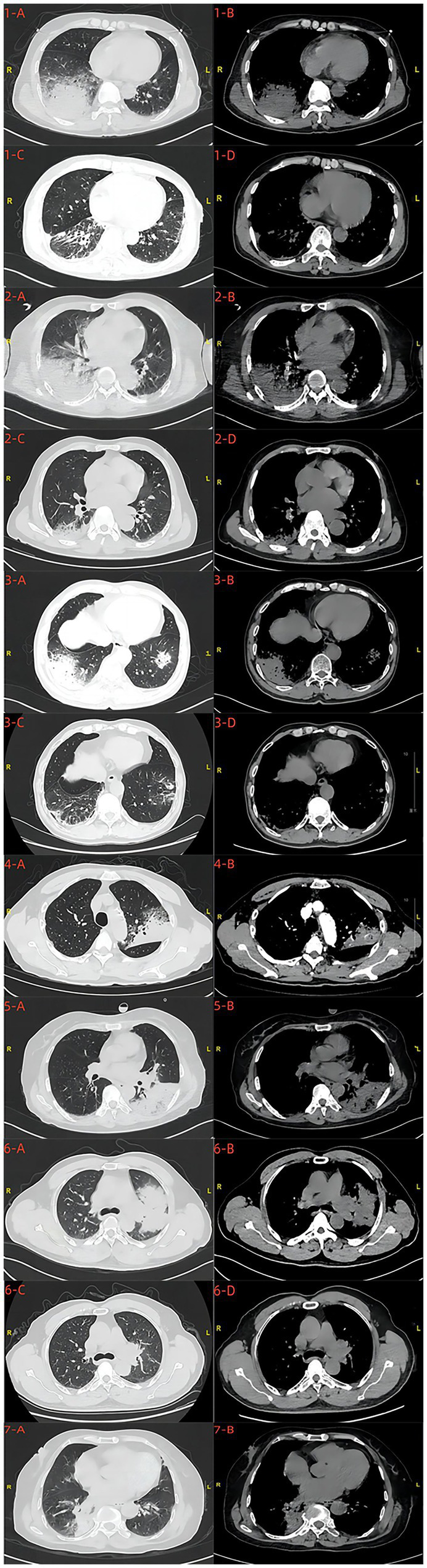
Chest CT scans before and after treatment 1-A,B,C,D; 2-A,B,C,D; 3-A,B,C,D; 6-A,B,C,D were, respectively, the chest CT scans of cases 1, 2, 3, and 6 patients before and after treatment. 4-A,B;5-A,B;7-A,B were, respectively, the chest CT scans of cases 4,5 and 7 patients before treatment.

Pneumonia and consolidation were found on chest CT scans in seven patients before treatment. Five patients presented with pleural effusion on pre-treatment chest CT scans. After treatment, significant improvement in pneumonia was observed in four patients based on post-treatment chest CT scans (Figure 1).

## 4 Discussion

*Chlamydia abortus*, characterized by its intracellular parasitic nature, is a pathogenic microorganism with a diameter ranging from approximately 200 nm to 500 nm and exhibits a negative Gram stain (12). Typically transmitted among poultry or mammals, extensive research has been conducted in the field of animal husbandry (13). Cases of human infection with *Chlamydia abortus* are rare, contrasting with the increasing incidence of *Chlamydia psittaci*, another zoonotic disease. Notably, two cases in our study involved co-infected with *Chlamydia psittaci.* This particular pathogen commonly infects poultry or mammals, and human transmission typically occurs through the inhalation of aerosols carrying the pathogen or contact with the feces, feathers, or carcasses of infected birds; recent reports also highlight instances of human-to-human transmission (14, 15).

As observed in our study’s Chinese cohort, all seven patients had a history of poultry exposure. The source of infection is presumed to be poultry carrying *Chlamydia abortus*, and humans transmission likely occurs through inhalation or contact with *Chlamydia abortus* living in poultry. While documented cases suggest contact transmission, particularly among infected pregnant women (9), human-to-human transmission remains unreported in other populations (16). This underscores the epidemiological significance of exposure history, emphasizing the importance of understanding patients’ occupations and assessing their exposure to poultry, birds, or mammals.

*Chlamydia,* like other intracellular parasitic microorganisms, relies on specific intracellular microenvironment for optimal growth. Studies have shown that *Chlamydia pneumoniae* can initiate infection in various organ cells through diverse biological activation pathways, leading to inflammatory responses, cellular oxidation, necrosis, and other manifestations (17, 18). The precise pathogenesis of *Chlamydia abortus* infection remains incompletely understood.

In this study, all seven patients had hyperthermia, six presented with respiratory symptoms, and two had digestive symptoms. Examination reports revealed a significant increase in the blood inflammation index of all patients, with six patients experiencing hyperventilation and hypoxemia, and four displaying liver function impairment. Chest CT scans before treatment showed pronounced lung inflammation, exudation, and pleural effusion. These cases illustrated that *Chlamydia abortus* can infect the human respiratory system, leading to disease characterized by a severe local inflammatory response in lung tissues and inflammatory manifestations in the bloodstream.

The inflammation and exudation of the lungs are marked, with lesions manifesting as consolidation and ground-glass changes. Consequently, patients present with hemoptysis such as cough, expectoration, and even hemoptysis, dyspnea, hyperventilation, hypoxemia, and other conditions. Comparative analysis with studies on *Chlamydia psittaci* pneumonia reveals striking similarities in terms of epidemiological contact history, symptoms, and chest CT scans. In addition, two patients in our study were co-infected with *Chlamydia psittaci.* A *s*tudy had shown that *Chlamydia psittaci* and *Chlamydia abortus* belong to the same genome of Chlamydia and their genomes are highly homologous, then they cause pulmonary infections with similar pathological mechanisms resulting in the same clinical presentation, and co-infection can even worsen the inflammatory response in lung (19). The pathophysiological changes of human infection with *Chlamydia abortus* need further investigation. A comprehensive understanding of the clinical features of *Chlamydia abortus* pneumonia can be gleaned through further summarization and analysis based on a larger body of case evidence.

**Figure 2:**
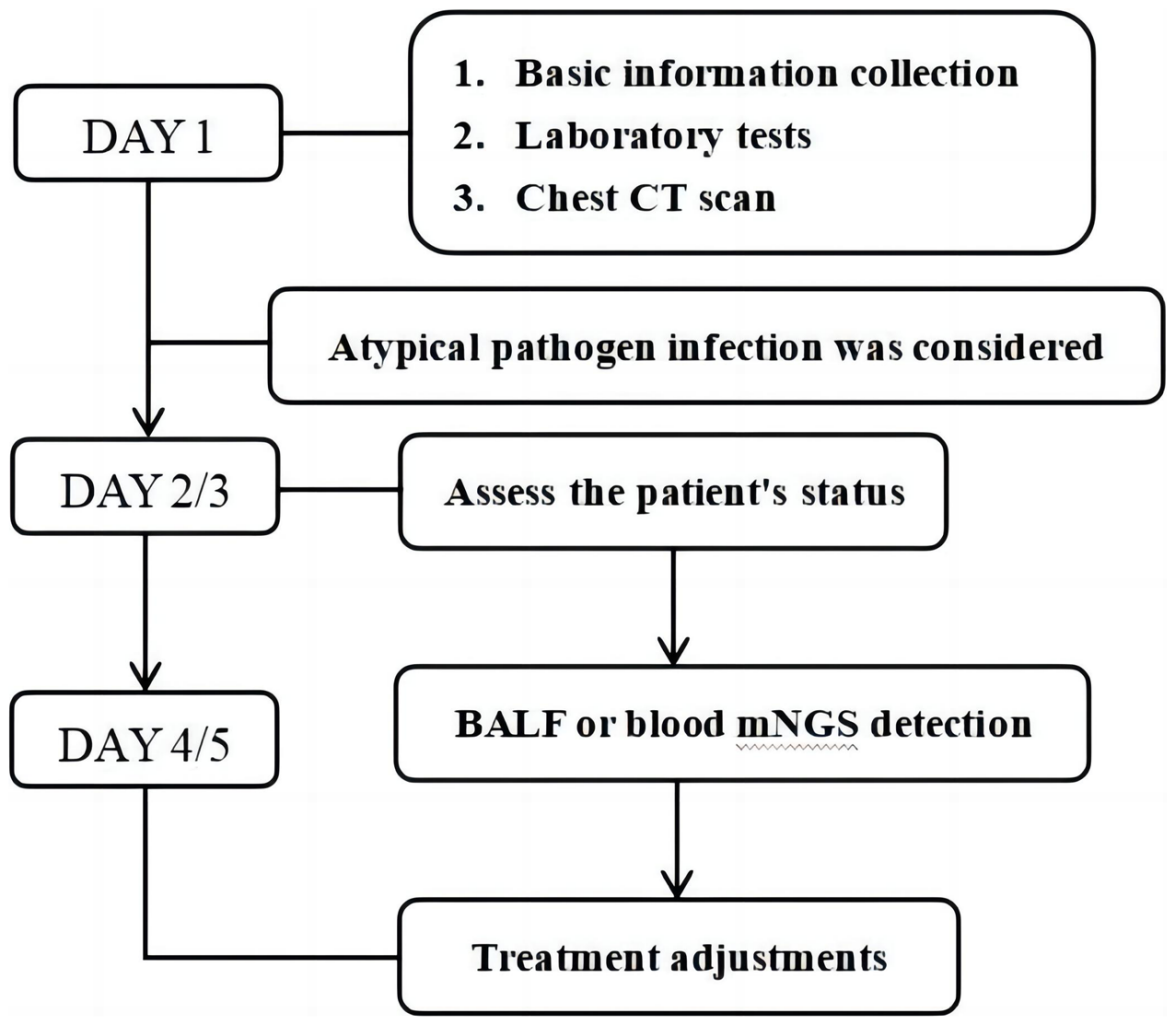
Flowchart of diagnosis and treatment of patients after admission.

*Chlamydia* infection is predominantly confirmed through testing for chlamydia nucleic acid or genetic sequencing (20). Currently, the clinical detection of *Chlamydia abortus* relies on mNGS, as traditional diagnostic methods are not adept at identifying this pathogen. High-throughput sequencing, a new type of microbial detection technology, is an advanced microbial detection technology known for its efficiency and cost-effectiveness. Sequence alignment facilitates the swift and accurate identification of microbial species (5). The increasing adoption of mNGS in clinical settings is primarily for the detection of challenging, rare and special infectious pathogens. This approach not only discerns the type and sequence of infected pathogens, but also offers substantial benefits to infected patients with poor effects by clinical empirical treatment. In clinical practice, we would encounter critical cases of rare pathogen infection, and mNGS could help us detect infectious pathogens at an early stage of the disease. All seven patients in this paper completed mNGS test at an early stage, which identified the infectious pathogen and allowed the patients to receive early treatment. On the first day after admission, we collected the patient’s basic information, completed laboratory tests and chest CT scan. If the patient was considered to be infected with an atypical pathogen, we assessed the patient’s condition on day 2/3 to complete BALF or blood mNGS test. The treatment regimen is then adjusted based on the results of mNGS (Figure 2). It enables rapid and accurate pathogens detection, guides antibiotic use in clinical practice, and holds significant promise in clinical applications.

In the past, limitations in detection methods hindered the identification and diagnosis of special pathogenic bacterial infections. The widespread use of mNGS has transformed this landscape, allowing for more informed anti-infective therapy decisions and preventing delays in patient conditions, as well as curbing antibiotic misuse. Furthermore, mNGS can detect various human specimens and identify different pathogens of co-infection. In our study, six patients underwent mNGS testing to detect *Chlamydia abortus* in BALF, while one patient (case 1) was diagnosed via mNGS testing using blood samples. Case 1 was found to be infected with *Chlamydia abortus*, co-infected with *Chlamydia psittaci* and *Pseudomonas aeruginosa.* Case 3 *and* 5 were co-infected with *Chlamydia psittaci.* Both *Chlamydia abortus* and *Chlamydia psittaci* belong to the genus *Chlamydia* and are often found parasitizing poultry. Human infection with these two pathogenic bacteria simultaneously underscores the potential for co-existence of *Chlamydia* with other bacteria, though the specific mechanisms require further exploration.

*Chlamydia* is an obligate intracellular parasitic organism that exhibits susceptibility to tetracyclines, macrolides, and quinolones, all of which interfere with intracellular DNA and protein production (21, 22). However, there is a notable high resistance to macrolides among the Chinese population, and the efficacy of quinolones in treating chlamydia infection is suboptimal, coupled with the risk of various drug-related side effects. At present, tetracyclines are the preferred choice for *Chlamydia abortus* infection in clinical practice. Tetracycline antibiotics, available in natural and semi-synthetic forms, are widely used in clinical practice, including Doxycycline, Minocycline, Omadacycline, etc. In this study, all seven patients were treated with Doxycycline, and six patients demonstrated significant improvements. And case 1 experienced relief of symptoms after transitioning to Omadacycline because of intermittent fever during Doxycycline treatment. The remaining six patients involved initial intravenous administration of Doxycycline followed by oral sequential Doxycycline therapy for about 10 to 14 days. Based on the patient’s symptoms and the results of some chest CT reexamination, Doxycycline had a positive therapeutic effect on the pneumonia caused by *Chlamydia abortus*. Doxycycline is widely used in our hospital and has demonstrated efficacy against infections caused by atypical pathogens, scrub typhus, and Q fever.

While there are reported cases in China highlighting the effectiveness of Minocycline against *Chlamydia psittaci* (23), our team has not utilized Minocycline anti-chlamydia therapy. However, the absence of specific clinical guidelines for guiding the anti-infective regimen and duration of tetracycline drugs in treating *Chlamydia psittaci* or *Chlamydia abortus*, underscores the need for additional real-world data to further elucidate these aspects.

This case series presents seven cases of *Chlamydia abortus* pneumonia diagnosed through mNGS testing. All seven patients exhibited an epidemiological history of poultry exposure, presented with fever accompanied by respiratory or digestive symptoms, demonstrated significantly elevated blood inflammation indicators, showed hyperventilation and hypoxemia in arterial blood gas analysis, and displayed changes on chest CT indicative of pulmonary inflammatory exudate, consolidation, and pleural effusion. Remarkably, the administration of tetracyclines led to a substantial improvement in symptoms for all seven patients. In summary, contact with poultry can lead to *Chlamydia abortus* infection in humans, resulting in severe pneumonia and systemic inflammation, a condition detectable through mNGS and responsive to tetracycline treatment. It is important to note the limitation in the follow-up status of the cases, as some patients did not return to the hospital for subsequent examination of chest and related inflammatory indicators assessments, hindering a comprehensive comparison of clinical data before and after treatment. Nevertheless, the study offers insights into the characteristics of *Chlamydia abortus* infection based on these seven cases, contributing valuable clinical evidence for future case management.

## Data availability statement

The original contributions presented in the study are included in the article/Supplementary material, further inquiries can be directed to the corresponding author.

## Ethics statement

The studies involving humans were approved by the Meizhou People’s Hospital Ethics Committee. The studies were conducted in accordance with the local legislation and institutional requirements. The participants provided their written informed consent to participate in this study.

## Author contributions

YW: Funding acquisition, Investigation, Project administration, Supervision, Writing – original draft, Writing – review & editing. YD: Project administration, Resources, Software, Supervision, Writing – original draft, Writing – review & editing. XS: Conceptualization, Data curation, Project administration, Resources, Writing – original draft, Writing – review & editing. ZZ: Methodology, Resources, Software, Validation, Writing – original draft, Writing – review & editing.
